# Supplementary material for: Rapid turnover of life-cycle-related genes in the brown algae
Source: Genome Biol. 2019 Feb 14;20:35. doi: 10.1186/s13059-019-1630-6 (PMC6374913; doi:10.1186/s13059-019-1630-6)
Supplement: Supplementary file 3 — Protein alignments, corresponding Gblocks html file and CDS sequences for the lineage conserved gametophyte-biased genes. (ZIP 2109 kb) [file 13059_2019_1630_MOESM3_ESM.zip › Con_GA_alignments/Ec-27_003990.aa_ali.fasta-gb.htm]

Ec-27\_003990.aa.fasta.trans.aa\_ali.fasta


## Gblocks 0.91b Results

Processed file: **Ec-27\_003990.aa.fasta.trans.aa\_ali.fasta**  
Number of sequences: **2**  
Alignment assumed to be: **Protein**  
New number of positions: **186** (selected positions are underlined in blue)

```
                         10        20        30        40        50        60
                 =========+=========+=========+=========+=========+=========+
Ec-27_003990     MQSLAIIPYSGGGAAEQPPCHRLFTLLESSESDAQVLRGAALPHGSFGGLPRNTRRLEEG
MSTRG.20713.1    FSVLHNLAWP------------HFVSQDVRDMSGSVRRGES-------GISAGSLR----
                                                                             


                         70        80        90       100       110       120
                 =========+=========+=========+=========+=========+=========+
Ec-27_003990     NVLPAGSGTQLGPALPPLNVEGGKERHQSKWMIVSKEQVEQTAEKMRDQAEKNKALETTV
MSTRG.20713.1    -------PQYGGPMLVARSNVRGVEERRSNWMIVSAEQVEHTAEKLRHQAEKNKVLESAI
                            #################################################


                        130       140       150       160       170       180
                 =========+=========+=========+=========+=========+=========+
Ec-27_003990     KIHKRERVLIYDAAMKQAAKRELEGVNEVKQVLERQQGMMRAEVDQEMQKISDLYQERCR
MSTRG.20713.1    EDHKRERILIYDAAMKQAAKREEEGINDAKLVLELEQSKMRSAVDQELRKIGDLYQERCR
                 ############################################################


                        190       200       210       220       230       240
                 =========+=========+=========+=========+=========+=========+
Ec-27_003990     DYATMVSDECDGEDSKAATDLRKDWARQKKTIDQLVRSGGEEDRQVSVIARAVVEQLQEE
MSTRG.20713.1    DYASMVSDECEASISKAASDLRKDWARQRKTIDELVQCGGEEDRQVSVIARAVMDQLREE
                 ############################################################


                        250       260       270       280       290       300
                 =========+=========+=========+=========+=========+=========+
Ec-27_003990     RRDEIASLRAAIYSDVKMEFESREVALRDTLESDFQADLDGQLLLAEIRHQKEMNRLRVE
MSTRG.20713.1    KMDDINALRADIYSDVKV------------------------------------------
                 #################                                           


                        310       320       330       340       350       360
                 =========+=========+=========+=========+=========+=========+
Ec-27_003990     LAGSEKPTTEAKTGEIVVYNPETPQCAGRGQRSSGTSSFTPSPSFSRRPLHKMNISEASG
MSTRG.20713.1    ------------------------MCA---------------------------------
                                                                             


                        370       380       390       400       410       420
                 =========+=========+=========+=========+=========+=========+
Ec-27_003990     GNSGADGVHVPTTPRGTQLGRKFPVFKTPVQKQIPGASNKKAVRPKEHPVRDSTRQPRNS
MSTRG.20713.1    ------------------------------------------------------------
                                                                             


                        430       440       450       460       470       480
                 =========+=========+=========+=========+=========+=========+
Ec-27_003990     SVGRHTEISTTTPIREQEDELDVGITSALMVSPTKAIYGVVTPSIGSEGEVSGEGSSRPK
MSTRG.20713.1    ----------------------------------HLVYTILTSLL---------------
                                                                             


                        490
                 =========+
Ec-27_003990     KRAKKLFSTE
MSTRG.20713.1    ---------F
```

```
Parameters used
Minimum Number Of Sequences For A Conserved Position: 2
Minimum Number Of Sequences For A Flanking Position: 2
Maximum Number Of Contiguous Nonconserved Positions: 8
Minimum Length Of A Block: 10
Allowed Gap Positions: None
Use Similarity Matrices: Yes
```

```
Flank positions of the 1 selected block(s)
Flanks: [72  257]  

New number of positions in Ec-27_003990.aa.fasta.trans.aa_ali.fasta-gb:  186  (37% of the original 490 positions)
```
